# Exercise, grit, and life satisfaction among Korean adolescents: a latent growth modeling analysis

**DOI:** 10.1186/s12889-024-18899-8

**Published:** 2024-05-23

**Authors:** Myeong-Hun Bae, Xiaoyu Zhang, Je-Seong Lee

**Affiliations:** 1 Department of Elementary Education, College of First, Korea National University of Education, Cheongju-si, Republic of Korea; 2 Department of Physical Education, Xinzhou Normal University, Xinzhou City, The People’s Republic of China; 3 Department of Sports Education, Gwangju National University of Education, Gwangju Metropolitan City, Republic of Korea

**Keywords:** Adolescents, Children, Exercise, Grit, Latent growth modeling, Life satisfaction

## Abstract

**Background:**

Life satisfaction among Korean students is declining substantially, and multifaceted improvement efforts are required.

**Methods:**

We analyzed longitudinal change trajectories for exercise, grit, and life satisfaction levels among Korean adolescents using latent growth modeling with longitudinal data from the Korean Children and Youth Panel Surveys of 2,142 students (male: 1,070, female: 1,072) from sixth grade (2020) through eighth grade (2022).

**Results:**

The model, which tracked linear changes in the students’ exercise, grit, and life satisfaction, showed consistent declines over three school years for all variables. We also identified a longitudinal causal relationship among exercise, grit, and life satisfaction. A higher grit intercept was associated with higher intercept for—and a partial mediating effect between—exercise and life satisfaction. The grit slope was positively related to the life satisfaction slope, and both the intercept and exercise slope had positive effects on life satisfaction. Moreover, grit had a longitudinal mediating effect between exercise and life satisfaction.

**Conclusions:**

We discuss the longitudinal change trajectories of exercise, grit, and life satisfaction, the causal and mediating effects among them, and the implications of the findings. These findings bolster our understanding of Korean adolescents’ life satisfaction and have practical significance for designing programs to improve their quality of life.

## Background

The term life satisfaction captures individuals’ overall cognitive assessments of their lives [1]. One’s satisfaction with life is dynamic; it changes in response to both internal and environmental factors and is particularly influenced by intrapersonal characteristics [2, 3]. Longitudinal studies in the UK and Germany have shown that life satisfaction declines sharply during adolescence [4]. Moreover, a recent report about children and adolescents in South Korea found that their life satisfaction declined as they ascended grade levels, representing the third-lowest score among the 38 Organization for Economic Cooperation and Development countries [5]. Declining life satisfaction among Korean students is a serious problem requiring multifaceted efforts to address the issue.

Exercise is a type of physical activity that is planned, structured, and repetitive, performed to improve or maintain physical fitness levels [6]. Physical activity provides significant benefits in the prevention, management, and treatment of chronic diseases [7–12]. Studies show that both physical activity and the lack of it (i.e., sedentary behavior) influence weight management, bone and cardiovascular health, fitness, and health-related quality of life in children and adolescents aged 5 to 17 years [13]. Studies of adults in various countries also report associations between physical activity and stress, anxiety, depression, and mood [14–17]; with studies among children and adolescents showing similar results. A systematic review of studies on children and adolescents, along with a Norwegian trial, reflects that physical activity is associated with mental and psychological health, including depression and self-esteem [18–21]. Physical activity is also positively associated with life satisfaction. A study of Spanish adolescents reports that regular physical activity is a mediator for improved life satisfaction [22]. In South Korea, exercise participation among adolescents has been reported to have a long-term impact on life satisfaction [23]. Further, this association extends beyond children and adolescents, with similar findings in studies of young, middle-aged, and older adults [24–27]. The literature also suggests that there are direct and indirect links among exercise, grit, and life satisfaction. According to Duckworth et al. [28], grit refers to one’s disposition regarding the “perseverance and passion for one’s long-term goals” (p. 1087). Grit has been reported to increase work engagement and decrease work burnout in adults [29, 30]. Increased work productivity due to a level of high grit has been shown to lead to higher work performance [31, 32]. In students, higher levels of grit have been reported to be associated with higher levels of motivation and academic engagement, and thus, higher levels of academic achievement [33]. Grit has also been shown to be positively related to positive psychological outcomes, such as well-being and life satisfaction, in both students and adults [34]. It has also been reported to relate to various indicators of physical health [35]. Although a review of the literature points to direct and indirect links among exercise, grit, and life satisfaction, these studies are mainly cross-sectional. Unfortunately, cross-sectional studies are limited in their ability to verify clear causal relationships among multiple factors due to the constraint of studying only one point in time. Additionally, because the results are derived based on a specific point in time, there are limitations in evaluating the effects of various factors. To overcome these limitations, our study repeatedly measured the characteristics of the same subjects and analyzed how these changed over time.

Specifically, our study fills a gap in the literature by investigating the longitudinal change trajectories of exercise, grit, and life satisfaction among Korean adolescents. We believe that our results can contribute to a better understanding of the factors influencing life satisfaction among Korean adolescents, ultimately helping us identify some practical measures that can improve their life satisfaction. To that end, we tested the following hypotheses.

### Hypothesis 1

Exercise, grit, and life satisfaction among Korean adolescents change linearly over time.

### Hypothesis 2

There is a longitudinal relationship between exercise, grit, and life satisfaction among Korean adolescents.

### Hypothesis 3

Grit has a longitudinal mediating effect between exercise and life satisfaction among Korean adolescents.

## Methods

### Study design and participants

Our study was based on data from the Korean Children and Youth Panel Surveys implemented by the Korea Youth Policy Research Institute [36]. We analyzed 2,607 elementary school students in the 6th grade cohort (ages 11 to 13 years) as of 2020, who were surveyed using stratified multistage cluster sampling. We analyzed the cohort’s 6th grade (2020), 7th grade (2021), and 8th grade (2022) responses, the last three years of available longitudinal data. The surveys were conducted face-to-face via household visits from August to November in each of the years. All participants and/or their legal guardians provided written informed consent and conducted according to the principles outlined in the Declaration of Helsinki. The survey collection process was approved by the Korea Youth Policy Research Institute Ethics Review Committee (approval numbers: 202,007-HR-Unique-016, 202,106-HR-Unique-011, and 202,206-HR-Unique-026-11). Our final analysis included 2,142 participants (male: 1,070, female: 1,072), excluding 465 who had at least one missing observation during the three-year study period (see Table 1 for sample details).

**Table 1:** Study participants (*n* = 2,142)

	Grade (year)	Valid (%)			System missing values (%)			Total (%)
		Male	Female	Total	Male	Female	Total	
First viewpoint	Grade 6th (2020), aged 11 years	1,211 (46.45)	1,200 (46.03)	2,411 (92.48)	102 (3.91)	94 (3.61)	196 (7.52)	2,607 (100.00)
Second viewpoint	Grade 7th (2021), aged 12 years	1,145 (43.92)	1,130 (43.35)	2,275 (87.27)	168 (6.44)	164 (6.29)	332 (12.73)	2,607 (100.00)
Third viewpoint	Grade 8th (2022), aged 13 years	1,155 (44.30)	1,156 (44.34)	2,311 (88.65)	158 (6.06)	138 (5.29)	296 (11.35)	2,607 (100.00)
Total		1,070 (41.04)	1,072 (41.12)	2,142 (82.16)	243 (9.32)	222 (8.52)	465 (17.84)	2,607 (100.00)

### Research measures

#### Exercise

Exercise was measured in three ways using content adapted from surveys conducted by the Korea Ministry of Gender Equality and Family Affairs and the Korean Youth Policy Research Institute [37, 38]: (a) hours of exercise and physical activity outside of school hours on weekdays during the past academic year; (b) hours of exercise and physical activity on weekends during the past academic year; and (c) hours of exercise to the point of sweating during the past week. Response options for exercise and physical activity time on weekdays and weekends were none (1 point), 0–30 min (2 points), 30 min to 1 h (3 points), 1–2 h (4 points), 2–3 h (5 points), 3–4 h (6 points), and > 4 h (7 points). Response options for the number of hours of sweat exercise in the past week were none (1 point), 1 h (2 points), 2 h (3 points), 3 h (4 points), and > 4 h (5 points). For statistical analysis, we converted the question about the number of hours of exercise in the past week to a seven-point scale, with Cronbach’s α 0.758 at the first viewpoint, 0.716 at the second viewpoint, and 0.731 at the third viewpoint.

### Grit

Grit was measured using the Children’s Grit Scale and included eight items [39, 40]. The following statements were presented: (a) “I find it difficult to concentrate when I start to do something and then think of something else”; (b) “I don’t get very frustrated when I get stuck trying to solve a problem and I let my frustration go faster than others”; (c) “I have been known to focus on a problem for a while and then lose interest”; (d) “I am a hard worker”; (e) “I often set goals, but I then set other goals before I reach them”; (f) “I find it difficult to keep working hard if something takes a long time to complete”; (g) “I finish what I start”; and (h) “I am diligent”. Responses to the questions were rated on a four-point Likert-type scale ranging from 1 (not at all) to 4 (very much). For statistical analysis, questions (a), (c), (e), and (f) were reverse scored, with higher scores indicating higher grit. The results of the reliability test showed that items (b) and (e) were unreliable; therefore, they were removed, leaving six items for the final analysis. Cronbach’s α was found to be acceptable at 0.711, 0.750, and 0.726 for the first, second, and third viewpoints, respectively.

### Life satisfaction

Life satisfaction was measured using five items adapted from the Life Satisfaction Scale of Diener et al. [41]: (a) “overall, my life is close to ideal”; (b) “the circumstances of my life are very good”; (c) “I am satisfied with my life”; (d) “I have achieved the important things I want in my life so far”; and (e) “if I could live my life again, I would change almost nothing”. Responses to the questions were on a four-point Likert-type scale ranging from 1 (not at all) to 4 (very much). Cronbach’s α was 0.810 at the first viewpoint, 0.822 at the second viewpoint, and 0.809 at the third viewpoint, indicating good levels of reliability.

### Statistical analysis

To test our hypotheses, we conducted the following analyses of longitudinal change patterns of the variables, causal relationships, and mediating effects. First, the characteristics of the study participants were checked using frequency analysis. Correlations and multicollinearity among the variables were checked through correlation analysis, and skewness and kurtosis were examined to confirm life satisfaction under normal distribution conditions. Second, to verify the longitudinal change patterns of the variables, we compared the unconditional and linear models for each variable and conducted a latent growth analysis to examine change patterns over time. Third, we conducted a multivariate latent growth modeling analysis to test the longitudinal causal relationship between the intercepts and slopes of the variables. Fourth, to test the longitudinal mediating effect of grit, we calculated the direct, indirect, and total effects of the multivariate latent growth model and performed bootstrapping. All analyses were conducted using SPSS and AMOS for Windows (version 24.0; IBM Corp., Armonk, NY, USA), with significance set at *p* < 0.05.

## Results

### The longitudinal change trajectories of the variables

#### Correlation and descriptive statistics

Our Pearson correlation analysis showed that the correlations between exercise and grit (0.062 < *r* < 0.221, *p* < 0.05), exercise and life satisfaction (0.060 < *r* < 0.118, *p* < 0.05), and grit and life satisfaction (0.200 < *r* < 0.423, *p* < 0.05) were all statistically significant (see Table 2). The overall distribution of the correlation coefficients among the variables ranged from 0.049 to 0.471, indicating no multicollinearity. Meanwhile, we found that the means of exercise, grit, and life satisfaction decreased over time, with skewness ranging from 0.096 to 0.475 in absolute value and kurtosis ranging from 0.253 to 1.233, satisfying the conditions of normal distribution, with skewness < ± 2.00 and kurtosis < ± 4.00 [40].

**Table 2:** Correlations and descriptive statistics of variables

	Exercise 1	Exercise 2	Exercise 3	Grit 1	Grit 2	Grit 3	Life satisfaction 1	Life satisfaction 2	Life satisfaction 3
Exercise 1	1.000								
Exercise 2	0.435**	1.000							
Exercise 3	0.377**	0.433**	1.000						
Grit 1	0.221**	0.076**	0.102**	1.000					
Grit 2	0.126**	0.137**	0.086**	0.471**	1.000				
Grit 3	0.103**	0.049*	0.062**	0.355**	0.463**	1.000			
Life satisfaction 1	0.107**	0.049*	0.067**	0.423**	0.246**	0.197**	1.000		
Life satisfaction 2	0.055*	0.073**	0.053*	0.244**	0.331**	0.200**	0.448**	1.000	
Life satisfaction 3	0.060**	0.066**	0.118*	0.212**	0.222**	0.324**	0.385**	0.430**	1.000
Mean	3.097	3.067	2.927	2.643	2.601	2.570	2.864	2.746	2.684
Standard deviation	1.380	1.320	1.323	0.420	0.389	0.409	0.529	0.539	0.534
Skewness	0.457	0.420	0.447	0.388	0.475	0.435	-0.316	-0.221	-0.096
Kurtosis	-0.349	-0.253	-0.297	0.757	1.233	1.073	0.745	0.588	0.515

1, first viewpoint; 2, second viewpoint; 3, third viewpoint

*p < 0.05, **p < 0.01, assessed through Pearson correlation analysis

### Change analysis of latent growth models

To estimate the change trajectories of exercise, grit, and life satisfaction, we compared a no-change model with a linear-change model, as shown in Table 3. To compare the fit of each latent growth model, we selected and examined indices centered on sample sensitivity and parsimony (the Turker–Lewis index (TLI), comparative fit index (CFI), and root mean square error of approximation (RMSEA)) [42–45]. For exercise, both the unchanged and linear models had good fit values (TLI > 0.90, CFI > 0.90, RMSEA < 0.08); however, the linear model had a better fit. For grit, the RMSEA value of the unchanged model did not meet the criterion (0.091), whereas the linear-change model had a better fit than the no-change model for all values. Regarding life satisfaction, the no-change model showed that all indices were below the criterion, whereas the linear-change model showed a good fit for all values. Thus, we selected the linear change model because the exercise, grit, and life satisfaction trajectories showed consistent changes at each of the three time points.

**Table 3:** Comparing model fit indices of change in exercise, grit, and life satisfaction

Variables	Model	χ2	df	*p*	Turker–Lewis index	Comparative fit index	Root mean square error of approximation
Exercise	No growth	39.270	4	< 0.001	0.974	0.965	0.064
	Linear	4.363	1	0.037	0.990	0.997	0.040
Grit	No growth	74.448	4	< 0.001	0.953	0.937	0.091
	Linear	0.556	1	0.456	1.001	1.000	0.000
Life satisfaction	No growth	215.945	4	0.000	0.847	0.796	0.157
	Linear	7.001	1	0.008	0.983	0.994	0.053

*p < 0.05, **p < 0.01, assessed through structural equation analysis

Table 4 shows the means, variances, covariances, and correlations of the linear change model for exercise, grit, and life satisfaction. The means of the intercepts and slopes of all three variables were significant (*p* < 0.001). This was especially notable for the slopes, which had negative intercepts and showed steadily decreasing trajectories over time, starting with the intercept. Exercise decreased by 0.086 points per year from 3.116 points, grit decreased by 0.036 points per year from 2.641 points, and life satisfaction decreased by 0.090 points per year from 2.856 points. The variance estimates showed that exercise slope (*p* < 0.05), life satisfaction (*p* < 0.01), and grit (*p* < 0.001) were significant, which suggests that there are individual differences in the intercepts and slopes of these three variables among Korean adolescents. The covariances of the three variables were significant at *p* < 0.05 for exercise, *p* < 0.001 for grit, and *p* < 0.01 for life satisfaction. Notably, the intercepts and slopes of the three variables were negatively correlated, indicating that the higher the exercise intercept, grit intercept, and life satisfaction intercept at baseline, the greater the decline over time. Therefore, Hypothesis 1, which predicted that exercise, grit, and life satisfaction among Korean adolescents change linearly over time, was supported.

**Table 4:** Mean, variance, covariance, and correlation of linear change models for exercise, grit, and life satisfaction

		Mean	Variance	Covariance	Correlation
Exercise	Intercept	3.116***	0.893***	-0.103*	-0.372
	Slope	-0.086***	0.085*		
Grit	Intercept	2.641***	0.093***	-0.016***	-0.435
	Slope	-0.036***	0.014***		
Life satisfaction	Intercept	2.856***	0.146***	-0.019**	-0.379
	Slope	-0.090***	0.017**		

*p < 0.05, **p < 0.01, ***p < 0.001, assessed through structural equation analysis

### Latent growth analysis: longitudinal causality of variables

To test the longitudinal causal relationship among the variables of exercise, grit, and life satisfaction based on the latent growth analysis of the change model, each variable was set as a linear change model; thus, nine paths were established for our research model. The test result of the research model was χ2 = 184.812, df = 21, *p* < 0.001, TLI = 0.930, CFI = 0.959, RMSEA = 0.060, indicating a good fit. Table 5 presents the path estimates for the latent growth model to illustrate the longitudinal causal relationship among exercise, grit, and life satisfaction. The paths from the exercise intercept to the grit intercept, the exercise intercept to the grit slope, the grit intercept to the life satisfaction intercept, and the grit slope to the life satisfaction slope were significant at the *p* < 0.001 level.

**Fig. 1:**
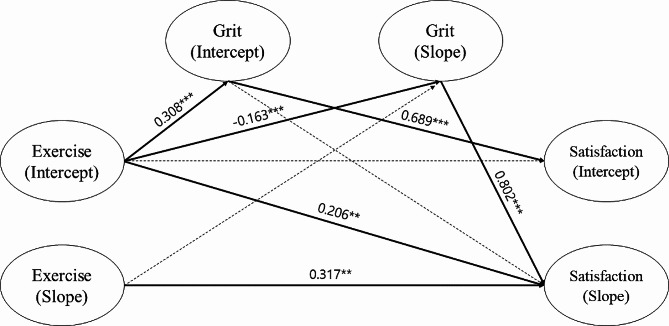
Path models of exercise, grit, and life satisfaction

For the path estimation, values of latent growth from the exercise intercept to life satisfaction slope and the exercise slope to the life satisfaction slope were significant at the *p* < 0.01 level; no significant values were found from the exercise slope to the grit slope, the grit intercept to the life satisfaction slope, or the exercise intercept to the life satisfaction intercept. The distribution of β values for the paths with statistical significance ranged from 0.206 to 0.802. Therefore, Hypothesis 2, which predicted that a longitudinal relationship exists among exercise, grit, and life satisfaction in Korean adolescents, was supported. Figure 1 presents the results of the path model based on the parameter estimates of our research model.

**Table 5:** Path estimation of latent growth model

Variables	B	β	Standard error	t
Exercise intercept → Grit intercept	0.101	0.308	0.011	8.925***
Exercise intercept → Grit slope	-0.024	-0.163	0.007	-3.569***
Exercise slope → Grit slope	0.013	0.032	0.023	0.541
Grit intercept → Life satisfaction intercept	0.823	0.689	0.049	16.966***
Grit intercept → Life satisfaction slope	-0.042	-0.103	0.032	-1.343
Grit slope → Life satisfaction slope	0.726	0.802	0.086	8.443***
Exercise intercept → Life satisfaction intercept	-0.019	-0.048	0.014	-1.312
Exercise intercept → Life satisfaction slope	0.028	0.206	0.010	2.896**
Exercise slope → Life satisfaction slope	0.113	0.317	0.038	3.020**

**p < 0.01, ***p < 0.001, assessed through path analysis

### The longitudinal mediating effects of grit

Table 6 presents the latent growth model values for the values of the direct, indirect, and total effects. The decomposition of the effects shows values of 0.212 for the indirect effect of the exercise intercept on the life satisfaction intercept, -0.162 for the indirect effect of the exercise intercept on the life satisfaction slope, and 0.026 for the indirect effect of the exercise slope on the life satisfaction slope.

**Table 6:** Direct, indirect, and total effects of latent growth models

Path	Direct effect	Indirect effect	Total effect
Exercise intercept → Grit intercept	0.308		0.308
Exercise intercept → Grit slope	-0.163		-0.163
Exercise intercept → Life satisfaction intercept	-0.048	0.212	0.164
Exercise intercept → Life satisfaction slope	0.206	-0.162	0.044
Exercise slope → Grit slope	0.032		0.032
Exercise slope → Life satisfaction slope	0.317	0.026	0.342
Grit intercept → Life satisfaction intercept	0.689		0.689
Grit intercept → Life satisfaction slope	-0.103		-0.103
Grit slope → Life satisfaction slope	0.802		0.802

*p < 0.05, **p < 0.01, assessed through effect decomposition analysis

To test the significance of the longitudinal mediating effect of grit between exercise and life satisfaction, we applied the bootstrap method [46]. We calculated the bootstrap results with 2,000 iterations at 95% confidence intervals to generate a distribution for the indirect effect and test the significance of the confidence interval for this. The results revealed significant values (*p* < 0.01) for the indirect effects of the exercise intercept on the life satisfaction intercept and the indirect effects of the exercise intercept on the life satisfaction slope among the students, and the bias-corrected 95% confidence intervals of the non-standardized coefficients of both paths did not contain zero, as shown in Table 7. Therefore, Hypothesis 3, which predicted that grit has a longitudinal mediating effect between exercise and life satisfaction among Korean adolescents, was supported.

**Table 7:** Mediation effects

Path			B	*p*	95% confidence intervals (bias-corrected)	
					Lower bounds	Upper bounds
Exercise intercept →	Grit intercept	→ Life satisfaction intercept	0.083	0.001	0.064	0.106
Exercise intercept →	Grit intercept and slope	→ Life satisfaction slope	-0.022	0.001	-0.035	-0.011
Exercise slope →	Grit slope	→ Life satisfaction slope	0.009	0.577	-0.030	0.52

Assessed through bootstrapping analysis

## Discussion

Our study applied latent growth models to analyze the longitudinal relationships among exercise, grit, and life satisfaction in Korean adolescents. Specifically, we examined the longitudinal change trajectories of Korean adolescents’ exercise, grit, and life satisfaction levels. We also examined the longitudinal causal relationships among these variables and the mediating effect of grit.

First, we found that a linear model depicting the change over time in Korean adolescents’ exercise, grit, and life satisfaction was the most appropriate for our objective. Notably, all three variables decreased consistently over time. These results are supported by a systematic review and meta-analysis that found that moderate and vigorous physical activity in children and adolescents declined with age [47]. A study of Spanish adolescents found that academic grit scores in the fourth grade of primary school decreased by the second year of middle school [48]. A study of adolescents in the UK and Germany reported the steepest decline in life satisfaction during adolescence [4].

A characteristic feature of the change trajectories in these studies was the presence of individual differences in the intercepts and slopes of the three variables. Farooq et al. [47] found that girls’ physical activity decreased significantly over time. The researchers used statistical analyses to validate these individual differences, although they did not identify the specific factors involved. Future studies that control demographic variables, such as sex and age, may provide additional insights on how to curb this decline. Notably, Farooq et al. also found that students with higher levels of exercise, grit, and life satisfaction at baseline experience a greater decline over time [47]. This phenomenon is commonly reported in longitudinal studies where students with higher intercept values have a relatively greater scope for decline [49].

Second, we found longitudinal causal relationships among exercise, grit, and life satisfaction among Korean adolescents. Specifically, a higher exercise intercept was associated with a higher grit intercept. This result aligns with that of a cross-sectional study of adolescents in South Korea, wherein exercise and physical activity participation were correlated with grit [50]. Likewise, a study of college students in the United States also found that grit increased with increasing amounts of physical activity, particularly vigorous physical activity [51]. Additionally, the exercise intercept was found to be negatively related to the grit slope. In other words, the higher the exercise intercept, the higher the grit intercept, and the higher the grit intercept, the greater the decline in grit. Therefore, although a positive correlation between exercise and grit was demonstrated, the greater decline in this higher intercept suggests a need to examine the factors that may inhibit grit.

A higher grit intercept was also associated with a higher life satisfaction intercept, with the grit slope positively correlating with the life satisfaction slope. Our findings are supported to some extent by studies of gifted adolescents in Hong Kong and university students in Turkey, which reported that grit is positively related to life satisfaction [52, 53]. These results suggest that the higher the student’s grit intercept, the more likely that the individual’s life satisfaction will be higher, and the greater the increase in grit, the greater the increase in life satisfaction, suggesting that grit is a factor worth considering in life satisfaction. Both the intercept and exercise slopes were shown to have positive effects on the life satisfaction slope. A study of 42 European and North American adolescents found that lower levels of physical activity were associated with lower levels of life satisfaction; a study conducted in Taiwan supported these findings, reporting a positive relationship between physical activity and life satisfaction [25, 54]. These results suggest that, given the trend of decreasing life satisfaction over time, the higher the exercise intercept, the smaller the decrease in life satisfaction over time. Furthermore, the greater the increase in exercise, the greater the increase in satisfaction, suggesting that exercise is a key factor to consider in predicting life satisfaction.

Third, we found that grit had a longitudinal mediating effect on exercise and life satisfaction among Korean adolescents. Although the exercise intercept’s effect on life satisfaction was not significant, the grit intercept had a significant mediating effect between the exercise intercept and the life satisfaction intercept, indicating a full mediation effect. Additionally, the exercise intercept had a significant effect on the life satisfaction slope and the intercept and slope in grit indicated a partial mediating effect between the exercise intercept and the slope in life satisfaction. In other words, higher levels of exercise participation do not directly predict higher levels of life satisfaction, but grit mediates the prediction of higher levels of life satisfaction. Furthermore, the higher the exercise intercept, the greater the decrease in life satisfaction mediated through grit. These findings are supported by a cross-sectional study of adolescents in South Korea that reported that exercise participation and physical activity had moderating effects on happiness via cooperation and friendship [50]. In addition, studies in Taiwan and Japan reported that adults’ lifestyles and participation in leisure sports influenced life satisfaction via grit, also supporting our findings [55, 56]. In summary, our results imply that exercise is an important factor influencing life satisfaction through the grit relationship.

### Limitations and practical applications

There are several limitations to our study worth noting. First, the data we analyzed were from a self-reported questionnaire. Although the measures included multiple items, this approach may have limitations in accurately measuring levels of exercise. There was also the potential for respondents to underreport problems in response to negative indicators. Second, although we used longitudinal data, our data were limited to the last three years. More precise results could be identified using a longer period; thus, further studies with continuous follow-up are warranted. In particular, if data were collected in the periods before, during, and after the onset of COVID-19, we could also assess how the global pandemic had affected adolescent lives. Third, we focused only on a cohort of school students; therefore, our study cannot be considered representative of all Korean youth. Hence, future studies should be conducted on adolescents of different ages. Additionally, the study only targeted Korean adolescents. Future research conducted in a variety of countries could contribute to a global understanding of exercise, grit, and life satisfaction. Fourth, our study was limited by the unidirectional relationship among exercise, grit, and life satisfaction. In future studies, an autoregressive cross-lagged model could be used to analyze the interactions among these variables. Additionally, research could be conducted that includes physical, psychological, and spiritual indicators among adolescents to help us understand them better and improve their lives. Finally, the latent growth model we used was limited to unconditional models. The application of a conditional model would enable researchers to track changes among the variables according to background factors, such as sex. Nevertheless, our study contributes to the literature by collecting and analyzing large-scale, nationally representative data.

Our study provides an important basis for understanding the lives of Korean students. The results show a significant decline in exercise, grit, and life satisfaction among these students. In other words, once Korean children reach adolescence, they have less perseverance and enthusiasm for physical activity and general academic performance. Their responses reflect that their lives are increasingly unhappy. Therefore, our results highlight the need to recognize the problems in the lives of Korean adolescents that require intervention.

Additionally, the study provides practical ideas for improving adolescent lives, as we tested factors affecting their life satisfaction. Specifically, we showed that exercise, grit, and life satisfaction were directly or indirectly related. The relevance of the intercepts is that they can be used to predict their cross-sectional change and the slopes can be used to determine their longitudinal relevance. In other words, exercise is a key factor that affects life satisfaction among adolescents, both directly and through the mediation of grit. Therefore, the major practical implication is that structured programs that increase exercise participation among adolescents can have a positive impact on grit and life satisfaction. Although our results confirm that youth’s exercise, grit, and life satisfaction decline over time, the findings also provide informed guidance for schools, communities, and families by verifying how exercise and grit can change life satisfaction. Our study paves the way for further research that considers long-term observations and investigates the association of other variables, thereby addressing our study’s limitations.

## Conclusions

In this study, we designed a latent growth model with exercise, grit, and life satisfaction as variables, based on three years of data collected from a Korean survey. We examined the longitudinal change trajectories of exercise, grit, and life satisfaction among Korean adolescents, the causal and mediating effects, and the implications of our results. Our study contributes to the literature by confirming the importance of exercise and grit intervention in adolescent life satisfaction and, thereby, provides a basis for designing programs that can improve adolescent quality of life.

## Data Availability

The author confirms that data supporting the findings of this study are available within the article. The datasets that support the findings of this study are available from the corresponding author on reasonable request.
